# Mu-Beta event-related (de)synchronization and EEG classification of left-right foot dorsiflexion kinaesthetic motor imagery for BCI

**DOI:** 10.1371/journal.pone.0230184

**Published:** 2020-03-17

**Authors:** Madiha Tariq, Pavel M. Trivailo, Milan Simic

**Affiliations:** School of Engineering, RMIT University, Melbourne, VIC, Australia; University of California San Diego, UNITED STATES

## Abstract

The left and right foot representation area is located within the interhemispheric fissure of the sensorimotor cortex and share spatial proximity. This makes it difficult to visualize the cortical lateralization of event-related (de)synchronization (ERD/ERS) during left and right foot motor imageries. The aim of this study is to investigate the possibility of using ERD/ERS in the *mu*, low *beta*, and high *beta* bandwidth, during left and right foot dorsiflexion kinaesthetic motor imageries (KMI), as unilateral control commands for a brain-computer interface (BCI). EEG was recorded from nine healthy participants during cue-based left-right foot dorsiflexion KMI tasks. The features were analysed for common average and bipolar references. With each reference, *mu* and *beta* band-power features were analysed using time–frequency (TF) maps, scalp topographies, and average time course for ERD/ERS. The cortical lateralization of ERD/ERS, during left and right foot KMI, was confirmed. Statistically significant features were classified using LDA, SVM, and KNN model, and evaluated using the area under ROC curves. An increase in high *beta* power following the end of KMI for both tasks was recorded, from right and left hemispheres, respectively, at the vertex. The single trial analysis and classification models resulted in high discrimination accuracies, i.e. maximum 83.4% for *beta* ERS, 79.1% for *beta* ERD, and 74.0% for *mu* ERD. With each model the features performed above the statistical chance level of 2-class discrimination for a BCI. Our findings indicate these features can evoke left-right differences in single EEG trials. This suggests that any BCI employing unilateral foot KMI can attain classification accuracy suitable for practical implementation. Given results stipulate the novel utilization of *mu* and *beta* as independent control features for discrimination of bilateral foot KMI in a BCI.

## 1. Introduction

People affected by neurological disorder, stroke, or spinal cord injury (SCI) necessitate a therapeutic goal of motor gait rehabilitation using assistive technologies [1, 2]. For lower-limb affectees, to re-gain the dorsiflexion of foot drop is vital [3–5]. The lost motor control functions could be emulated by inducing neuroplasticity using a brain-computer interface (BCI) system [6]. BCI provides an alternative neuropathway that translates human brain activities into commands for controlling external devices or prostheses [6, 7].

BCIs that use EEG features such as oscillatory/sensorimotor rhythm (SMR) are recorded over the somatic sensorimotor cortex. SMR are concentrated in the *alpha* (*mu*) (7–12 Hz), *beta* (13–35 Hz), and gamma (>36 Hz) frequency bands [8, 9]. BCIs have successfully deployed SMR to identify any changes related to the physical movement (motor execution, ME) or imagination of movement (motor imagery, MI) of any limb [10]. This is because an increase in the corticomotor excitability is involved during MI and ME of limb movement which is both muscle-specific and temporally modulated [11]. Both the execution and imagery tasks have been used in experiments, because the ME and MI implicate overlapping neural structures within the central nervous system [11]. However, from literature, MI tasks have been preferred over ME ones, to avoid any possibility of proprioceptive feedback. The MI is a covert cognitive process, where the user makes a kinaesthetic imagination of his/her own limb movement without any muscular intervention, also called kinaesthetic motor imagery (KMI) [1, 12].

Each limb movement elicits a unique pattern in the SMR *mu* and *beta* features [9]. These patterns are reflected in the form of either a power decrease termed event-related desynchronization (ERD) that correlate to movement preparation [13], or an increase in power termed event-related synchronization (ERS) associated to resting/idling state, or an inhibitory state [14, 15]. Increased idling might correspond to reduced inhibition of downstream, movement related neurons. The cortical localization of ERD/ERS patterns is due to the somatotopic arrangement of the motor cortex. The upper limbs e.g. hand area representation is on the mantle of the cortex, followed by lateralization [16], that makes the spatial discrimination between left and right movement prominent compared to lower limbs. The right-left hand ME or KMI *mu* ERD correlate to the bilateral hand area (C3 and C4 electrode positions) of the sensorimotor cortex with evident contralateral dominance compared to ipsilateral side [17, 18]. These contralateral and ipsilateral differences in *mu* ERD have been classified by BCI to be used as control features for operating external devices [19–21].

On the contrary, right-left lower-limb ME or KMI tasks are not extensively deployed due to the close location of left-right lower-limbs’ areas to each other. The foot motor area is situated deep within the interhemispheric fissure of the sensorimotor cortex that makes the left and right foot ME or KMI difficult to be spatially discriminated since they produce nearly identical EEG patterns [16]. Therefore, we can find studies where general foot KMI-based BCIs deploy feet KMI as one feature without discriminating the left-right side [22, 23]. However, studies available on the left-right discrimination of foot KMI, proposed the *mu* ERD and *beta* ERS/rebound (post task completion), as possible EEG features for classification [7, 17, 24, 25]; where the ERD/ERS patterns generate at the vertex [24]. According to [18], if a BCI user exhibits a slight left–right difference, the differences could be enhanced, and improve the control accuracy of a BCI via visual feedback. Besides aforementioned features, the possibility to research *mu* ERS as a new feature for classification of left-right foot KMI task is less due to its limited frequency bandwidth, but *beta* ERD still has a significant margin to be explored due to its wider frequency range. Hashimoto et al. [17] proposed a bipolar referenced ERD–ERS lateralisation enhancement, resulting in a two-class (left vs. right foot) classification accuracy of 81.6% in synchronous mode for one out of nine subjects. However, this was not the case with the remaining eight subjects, with an average classification accuracy of 69.3% for all subjects. The low average classification accuracy yields the possibility to deploy other methodology designs for higher discrimination accuracy. Further analysis of ERD/ERS in the *mu* and *beta* frequencies could provide more informative feature vector.

The incorporation of machine learning algorithms in the bilateral left-right foot classification is limited to linear discriminant analysis (LDA) or to the support vector machine in case of unilateral foot KMI [17, 25]. Careful selection of a new algorithm based on the size of the feature vector and its dimensions is required. For BCI systems that employ low dimensional feature vectors, the KNN algorithm can prove to be efficient [26].

Present study investigated the possibility to exploit the spectral, spatial and temporal EEG features in the range of 7 to 35 Hz, i.e. *mu* ERD, *beta* ERD, and *beta* ERS (post movement). The aim was to propose a novel methodology for analysis and discrimination of unilateral foot KMI using the common average and bipolar references, and comparing the features resulting from each reference. We proposed three classification models i.e. LDA, SVM, and KNN, to assess their performance against the statistical chance level, followed by the evaluation of developed method and multiple comparison corrections. Such features could be useful in BCI applications where more than one output necessitate in a system. The study directs to the utilization of three independent control features that could be used within one BCI system.

## 2. Methods

### 2.1 Participants

This study involved nine healthy participants, aged between 21–28 years, taking part in the experiment voluntarily. All participants submitted a written consent, i.e. they signed a participant consent form to engage in the study. None of them had any history of neurological disorder. Participants had no prior BCI experience. For this research, the ethics committee granted the approval, i.e. the RMIT College Human Ethics Advisory Network (CHEAN) Melbourne, Australia.

Participants were directed to sit on a comfortable chair and watch a monitor screen (17”) placed in front of them, at a distance of approximately 1.5 m. In order to avoid the possibility of proprioceptive signals induced due to muscle movement, a flat wooden surface was placed underneath the feet of participants. This way, both legs were loosely fixed. That allowed the knees to flex at 60° from full extension position, and ankles at the neutral position. During the experiment, participants were asked to dorsiflex their foot approximately 25° for 1 second, in accordance with the nominal walking gait measurements [27].

### 2.2 Cortical activity recording

**Fig 1:**
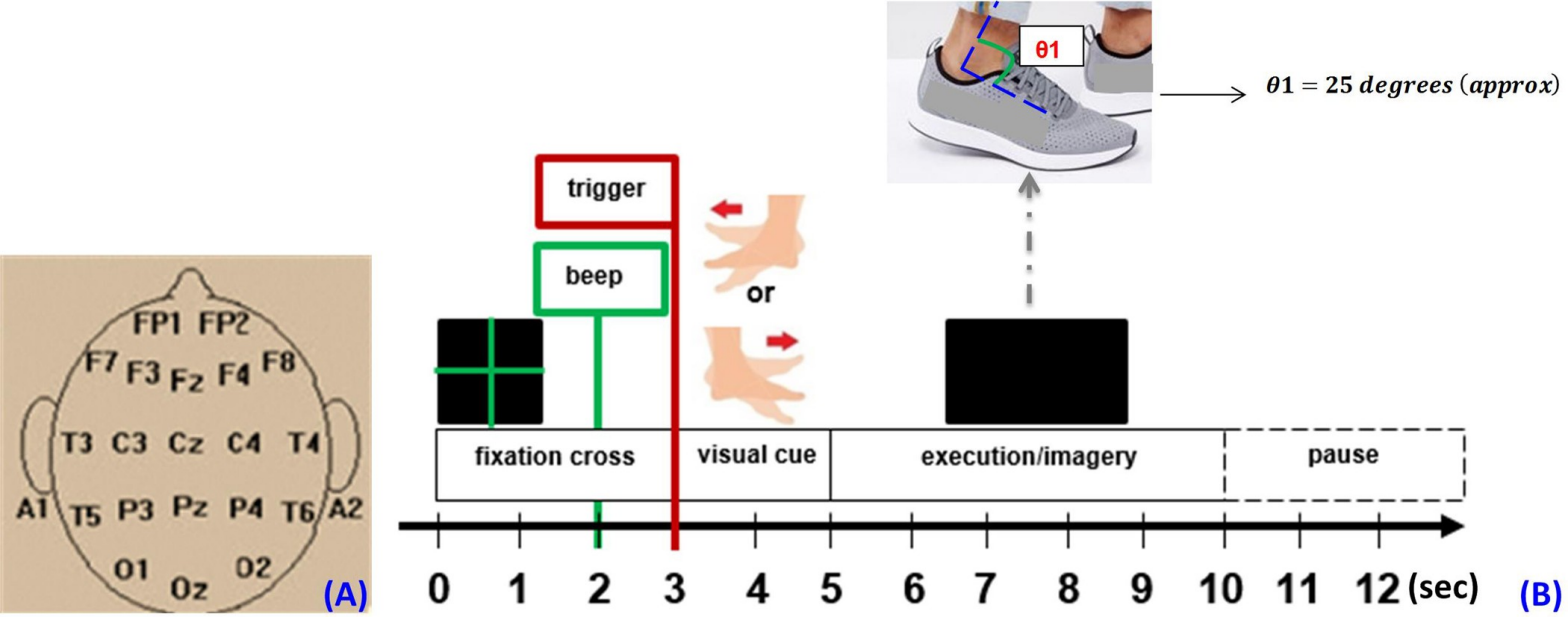
(A) EEG electrode/channel locations. (B) Experimental protocol for foot kinaesthetic motor imagery tasks, with cue timings expressed in seconds, during one trial.

EEG signals were recorded using the neurofeedback BrainMaster Discovery 24E amplifier (BrainMaster Technologies Inc., Bedford, USA). EEG was referenced to the linked earlobes A1 and A2 and recorded from 19 scalp electrodes. In order to acquire signals from the motor cortex, an electrocap with electrodes (C3, C4, Cz, F3, F4, F7, F8, Fz, FP1, FP2, O1, O2, P3, P4, Pz, T3, T4, T5, T6) mounted in and positioned according to the international 10–20 system [28], was used, as shown in Fig 1A. Monopolar EEG was amplified and band-pass filtered in the frequency range of 1–100 Hz. All channels were sampled at 256 Hz, quantised with 24-bit resolution. Ground electrode was positioned near the forehead of the participants. The experimental protocol was designed using OpenViBE designer tool that comes with integrated feature boxes [9, 29, 30].

After recording, EEG signals were processed. We converted the EEG signals into reference-free forms to analyse results from two different EEG derivations, i.e. common average and bipolar reference methods. The common average reference method used all electrodes as an identical reference electrode. Whereas for the bipolar method, the monopolar electrodes which were used for feature extraction, were limited to those near the vertex, i.e. voltage differences were transversely measured at two channels C3-Cz and Cz-C4, to emphasize the left-right cortical differences elicited in *mu*, low *beta*, and high *beta* for analysis.

### 2.3 Foot motor tasks

The experiment consisted of four cue-based sessions without feedback. Each session comprised of 40 trials, with 20 trials for left foot and 20 trials for right foot KMI in a random order. Before the four cue-based KMI sessions, a practice session was conducted, wherein participants performed a motor execution (ME) session of the same task in order to practice for measurement trials. During ME, participants were instructed to dorsiflex the foot approximately 25° maintained for 1 second (nominal walking gait) at each cue, Fig 1B. Following practice, the KMI sessions were conducted.

Each trial began with the presentation of a green fixation cross on screen for 3 seconds, used as reference period for processing of epochs. One second long audio beep stimulus, precisely before the visual cue display, was incorporated in the first trial only. This was to alert the participant about the beginning of the experimental trial, see Fig 1B. Next, the visual cue of 2 seconds length was displayed followed by a 5 seconds long blank screen to perform the related task (imagery), without blinking eyes. This made a total of 10 seconds for each trial. This was followed by random pause intervals of 1.5–3.5 seconds at the end of the trials, to prevent fatigue. The visual cues in each trial reflected either the left or right foot dorsiflexion image with an arrow pointing in the respective direction. Both visual cues were displayed in a random order to avoid possibility of any adaptation.

### 2.4 Time-frequency analysis and topography of ERD/ERS

In this study, we analysed both the ERD and ERS for each SMR i.e. *mu* (7–12 Hz), low *beta* (13–24 Hz) and high *beta* (25–35 Hz) [31]. An internal or external paced event results in the generation of an ERD/ERS, which is time-locked but not phase-locked to the event (induced) [32]. The decrease in percentage power or synchrony of the underlying neurons is termed ERD, while its increase is called ERS, with respect to a reference period [33].

**Fig 2:**
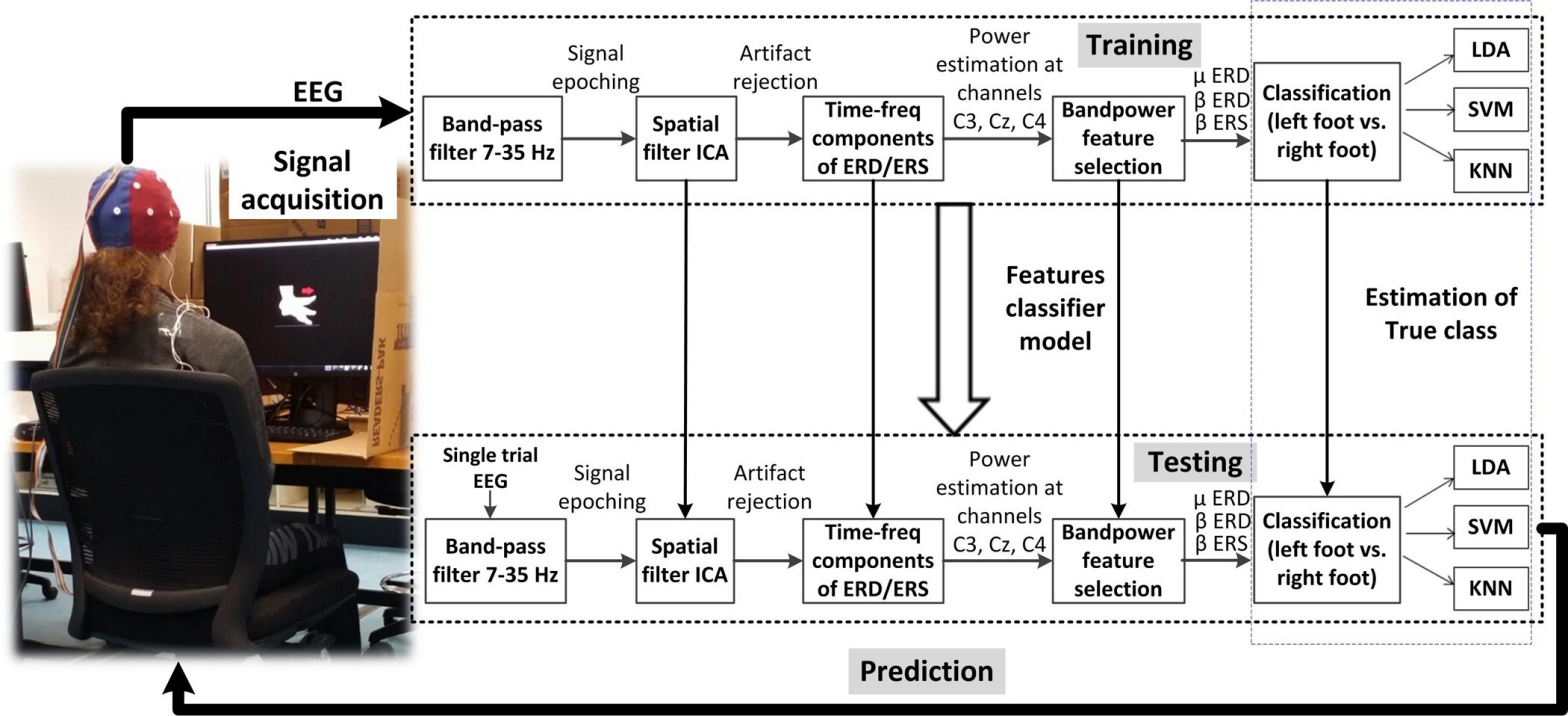
Band-power feature decoder with classifier training and testing in one-fold of the cross-validation.

The EEG was first converted to reference-free form by using the common average reference method first and then the bipolar reference. Data was pre-processed using finite impulse response (FIR) bandpass filter (implemented in EEGLAB [34]) with a low-cut frequency of 7 Hz and high-cut frequency of 35 Hz for capturing *mu* and *beta* rhythms, as shown in Fig 2. These frequency bands contain most informative features about the limb movements for classification of feature vectors [35].

Following this, epoching of the 40 trials (10 seconds) was done separately (class-wise), i.e. 20 left foot KMI and 20 right foot KMI epochs. Each extracted trial included the period of -3 to 0 seconds prior to the cue onset, used as reference period (baseline). The time window of cue for task performance was kept 5 seconds since dominant ERS occurs following movement offset. The processing of recorded data was based on single-trial EEG signals. Here the single-trial EEG refers to the EEG signals recorded during the KMI task (left vs. right) of one single trial. Based on [36], there is approximately 5% probability of any chance of EMG contamination in *beta* frequency band. In order to remove any ocular or EMG artifact from the EEG signal, we employed the independent component analysis (ICA) in the EEGLAB environment [34], as shown in Fig 2. Spatial filter in single-trial analysis, improves the signal-to-noise ratio [37, 38]. ICA represents the spatial weight structure or filter, that when applied to channels’ EEG activity, generates statistically independent projections.

Following this, artifacts (such as EMG contamination due to muscle movement) were filtered out, using *‘reject components by map’* (EEGLAB function). To analyse the difference between left and right foot dorsiflexion in the spectral and temporal domains, the time-frequency (TF) features that represent the subject-specific ERD/ERS patterns in the signal were obtained with channels C3, Cz, and C4 (which are subsequently used for machine learning). Using inter-trial variance (ITV) method [39], a script was developed in which the filtered average event-related potential (ERP) were subtracted from stimulus-locked single filtered EEG trials (class-wise), to overcome masking of induced activities. The samples were squared and sampled to estimate the power change in each frequency band, as presented in [14, 40]. For every trial, a wavelet coefficient matrix was computed with 100 time samples and 3 separate frequency bins (7–12 Hz for *mu*, 13–24 Hz for low *beta* and 25–35 Hz for high *beta*) for the component signal.

### 2.5 Feature extraction

For *mu* and *beta* band-power (BP) feature extraction from channels C3, Cz, and C4, associated to the sensorimotor cortex for motor imagery [24], an algorithm was developed, Fig 2. The common average reference and bipolar references were taken into account separately, for analysing the most significant BP decrease, or increase, during each of the left and right KMI task. The power spectrum density of each EEG epoch, which was determined using TF analysis, was calculated. For BP calculations, let *x*(*t*_0_,*t*_*f*_) be a single-trial EEG signal epoch within the time interval(*t*_0_-*t*_*f*_), where *t*_0_ and *t*_*f*_ are the time points in seconds satisfying the condition for task performance duration i.e. 2 ≤ *t*_0_ < *t*_*f*_ ≤ 7. For a specific frequency band i.e. *mu*, low *beta*, or high *beta*, the percentage power change y for left or right KMI EEG epoch *x*(*t*_0_,*t*_*f*_) is given as:

$$y\left(t_{0},t_{f}\right)=\frac{BP_{MI}\left(t_{0},t_{f}\right)-{\bar{BP}}_{baseline}\left(t_{0},t_{f}\right)}{{\bar{BP}}_{baseline}\left(t_{0},t_{f}\right)}$$ (1)

In Eq 1, *BP*_*MI*_(*t*_0_, *t*_*f*_) is the band-power of *x*(*t*_0_,*t*_*f*_); and ${\bar{BP}}_{baseline}\left(t_{0},t_{f}\right)$ is the mean band-power of the baseline prior to cue onset EEG epochs within the same time interval, given as:

$${\bar{BP}}_{baseline}\left(t_{0},t_{f}\right)=\frac{1}{N}\overset{N}{\underset{i=1}{\sum }}BP_{baseline}\left(t_{0},t_{f}\right)$$ (2)

**Fig 3:**
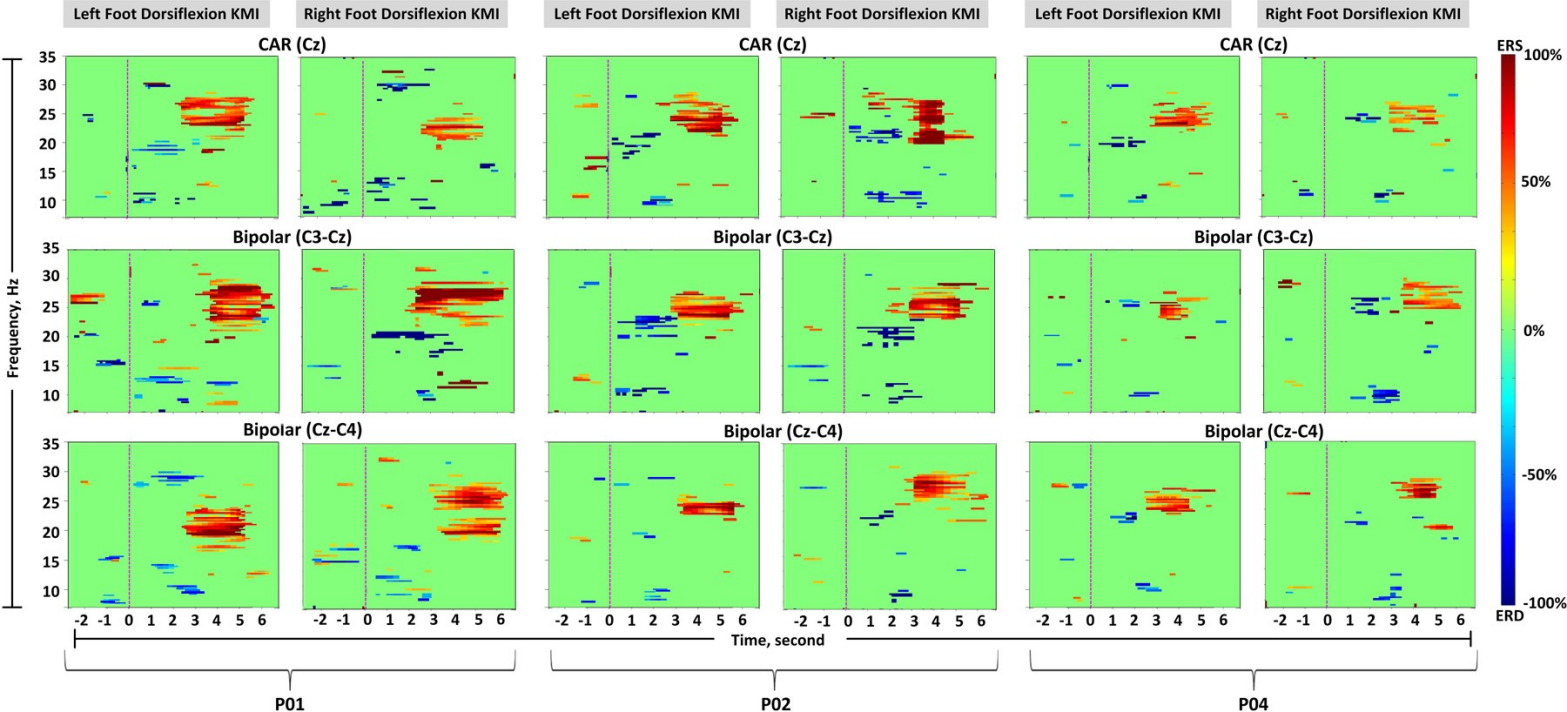
Time-frequency maps (participant 1, 2, and 4). Common average reference channel Cz, and two bipolar channels, C3-Cz and Cz-C4, are shown. The left columns show left foot dorsiflexion kinaesthetic motor imagery (KMI), and the right columns show right foot dorsiflexion KMI. Significant (*p* < 0.05) band-power changes are shown during the trial period of -3 to 7 s. The pink dotted line indicates the beginning of KMI.

In Eq 2, *N* = 20, as twenty baseline EEG signals were taken as reference for twenty left epochs (KMI cues), similarly *N* = 20 for twenty right epochs with each participant. The significance of deviations from baseline power was evaluated using bootstrap-t statistical method [41], with confidence interval of 95% (*p* < 0.05). A substitution for data distribution was created by selecting spectral estimates for each trial from the randomly selected latency windows in the assigned epoch baseline i.e. prior to the stimulus onset, followed by their averaging. After repeating this process several times (default: N = 200) a substitute ‘baseline’ amplitude distribution was generated whose specified percentiles were then taken as significant thresholds i.e. significant ERD and ERS features, as shown in TF maps, given in Fig 3.

#### 2.5.1 Test-statistic and family-wise error rate correction for multiple comparisons of ERD and ERS

For statistic evaluation and comparison of features, two independent samples *t-test* were conducted on the two groups (left foot KMI and right foot KMI) of each feature for channels C3, Cz, and C4, across participants. Table 1 shows the actual test-statistic values for common average reference and bipolar reference features. These *p*-values were used for multiple comparisons, to identify statistically significant features.

**Table 1:** Test-statistic values of adjusted *p-*values with 95% confidence interval, by comparing left foot KMI vs. right foot KMI, for common average and bipolar references.

Feature	Source	Degrees of freedom, df	Common average reference (Left Vs. Right)						Bipolar reference (Left Vs. Right)			
			C3		Cz		C4		C3-Cz		Cz-C4	
			*t*	*p*	*t*	*p*	*t*	*p*	*t*	*p*	*t*	*P*
Mu ERD	Between feature groups	8	7.020	0.001	1.100	0.012	3.122	0.003	3.131	0.010	3.970	0.014
	Within feature groups	49										
Beta ERD	Between feature groups	8	5.415	0.004	1.176	0.013	1.846	0.002	1.243	0.016	2.757	0.013
	Within feature groups	189										
Beta ERS	Between feature groups	8	9.605	0.001	3.2739	0.015	6.413	0.015	7.069	0.006	14.206	0.003
	Within feature groups	189										

For multiple comparison corrections/*p*-values adjustment, Bonferroni correction was used. The scheme used for each channel is displayed:

**Figure:**
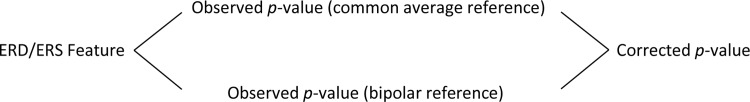


The observed *p*-values obtained from each reference were corrected for *mu* and *beta* ERD/ERS, respectively. Eq 3 is used to calculate the Bonferroni correction [42].

$$\frac{\alpha_{PC}}{c}$$ (3)

where α_PC_ is the specified per comparison error rate, and *c* is the number of comparisons performed. Here, α_PC_=0.05, with three statistical analyses conducted on the same sample of data, *c* = 3. Any observed *p-*value less than the corrected *p-*value i.e., 0.017 were declared statistically significant, as seen in Table 1. With each feature, the comparisons were done for one channel from common average reference and two from bipolar channels, given as:

- C3, and C3-Cz, Cz-C4
- Cz, and C3-Cz, Cz-C4
- C4, and C3-Cz, Cz-C4

Table 1 reflects the statistical test results i.e. the degrees of freedom, *t-*values and adjusted *p*-values, with Bonferroni correction. The test was conducted on time-windows starting from visual cue presentation (0-2s) to KMI task performance (2-7s), where peak latencies occur (with respect to cue-onset). Based on the adjusted *p*-values, the 2-D topographic ERD/ERS scalp maps were constructed for each participant, using the average ERD/ERS values in the most reactive frequency bands on all channels by using ‘topoplot’ (EEGLAB function).

#### 2.5.2 Individual peak latencies

With both references, the significant features with adjusted *p*-values were detected at a specific latency from cue-onset for each participant. They occur in the range of 7–12 Hz for *mu* ERD and between 13–35 Hz for *beta* ERD/ERS, as shown in Table 2. It reflects the individual peak latencies for left and right KMI task, in the respective frequency bands.

**Table 2:** Individual peak latencies from cue-onset for significant *mu* ERD, *beta* ERD, and *beta* ERS, using common average reference (CAR) and bipolar reference (BIP), across participants.

Participant	Mu ERD (7–12 Hz)				Beta ERD (13–35 Hz)				Beta ERS (13–35 Hz)			
	Latency from left- cue (s)		Latency from right- cue (s)		Latency from left- cue (s)		Latency from right- cue (s)		Latency from left- cue (s)		Latency from right- cue (s)	
	CAR	BIP	CAR	BIP	CAR	BIP	CAR	BIP	CAR	BIP	CAR	BIP
P1	2.62	2.75	2.80	2.90	1.90	1.80	1.82	2.00	4.20	4.65	4.50	4.50
P2	2.83	2.32	2.73	1.90	2.11	2.25	1.95	2.10	3.85	3.95	4.05	3.90
P3	1.80	2.10	2.22	2.10	1.62	2.15	1.78	1.95	2.80	2.75	3.15	3.05
P4	2.64	2.60	2.75	3.00	2.23	1.75	2.15	1.90	3.80	2.75	3.65	3.58
P5	2.70	2.65	2.42	2.55	1.75	1.88	2.17	1.65	2.65	2.88	2.60	2.58
P6	1.71	1.85	1.90	2.10	1.90	2.00	1.78	1.85	2.89	3.05	2.79	2.85
P7	2.00	2.10	2.15	2.60	1.81	1.74	1.77	1.75	3.52	3.65	3.55	3.80
P8	2.31	2.20	2.62	2.52	1.94	2.10	1.65	1.60	3.35	3.30	3.30	3.55
P9	2.45	2.15	2.20	2.52	1.60	1.70	2.15	2.25	3.55	3.78	3.60	3.58
Mean	2.34	2.30	2.42	2.47	1.87	1.93	1.91	1.89	3.40	3.42	3.50	3.58
S.D.	0.41	0.30	0.32	0.37	0.21	0.20	0.20	0.21	0.53	0.65	0.60	0.59

### 2.6 Evaluation of foot kinaesthetic motor imagery classification

As this study is based on synchronous mode BCI, the classical LDA was used to measure the classification accuracy for discrimination of left and right foot KMI [43, 44]. However, in order to improve the classification accuracy, linear-SVM and KNN algorithms were employed in addition to LDA [26].

With each classifier model, cross validation was used to estimate the optimal parameters for a classifier and avoid overfitting the classifier to the training data [45]. The *k*-fold cross validation is used for estimating the true performance of machine learning models used in the study. We partitioned the training data set into *k* folds of equal size, then using k—1 part as a training set and checked the classification rate on the one remaining part (testing set). This is repeated for *k* times (folds). Finally the accuracy on each fold is estimated by calculating the average of *k* classification rates obtained for *k* testing sets [46]. Feature scaling (standardization) was performed on the training set that transferred over to the test set. With each model, the dataset was randomized thirty times and each time divided into five folds (*k* = 5). The training set for each participant consisted of 190 trials, i.e. 95 trials for each KMI task. This means each test validation set consisted of 38 trials for each KMI task. Consequently, the weight vectors and classification accuracies of 5-folds were averaged. The mean and standard deviation of each classifier output was determined.

Linear SVM model used a linear kernel function [47]. The parameter *C*, which is the regularization parameter for controlling trade-off between attaining a low training and a low testing error, was verified for a range of values using MATLAB script. An optimal setting of *C* = 10 for the three models resulted in peak classification accuracies. In case of KNN, we used the weighted KNN method, and took into account *k* = 10, where *k* is the number of nearest neighbors, we wish to take vote from in the sample data [26]. The distance metric was Euclidean and the distance weight was squared inverse. In order to avoid overfitting, we assessed the training and validation error rate respectively, using different values of *k* and obtained the optimized result at *k* = 10 with each model and used it for prediction accuracy.

#### 2.6.1 Area under receiver-operator characteristic curve

To evaluate the performance of the classifiers, the receiver operating characteristic (ROC) curves were utilized, as reported in [17]. When using the ROC curve as an evaluation tool, the area under curve (AUC) defines the performance of the detector. It indicates how much the model is capable of distinguishing between classes. In ROC, along the x-axis is the sensitivity, called true positive rate (TPR), given as:

$$TPR=\frac{TP}{TP+FN},$$ (4)

where *TP* is the number of true positives and *FN* is the number of false negatives. Along the y-axis is 1-specificity, also termed the false positive rate (*FPR*), given as:

$$FPR=1-Specificity=\frac{FP}{TN+FP},$$ (5)

where *FP* is the number of false positives and *TN* is the number of true negatives. Ideally, the area under the ROC, i.e. the AUC = 1 indicates that there is 100% chance that the model will be able to distinguish between classes. For each model we calculated the *TPR* and *FPR* to obtain AUC-ROC in percentage across participants.

#### 2.6.2 False discovery rate correction

For comparing the performance of three independent classifier models i.e. controlling the expected proportion of the rejected null hypotheses which are erroneously rejected, we implemented false discovery rate (FDR) correction, using the Benjamini-Hochberg procedure [48]. For Table 3, Eq 6, defines the FDR [49].

**Table 3:** Errors in multiple testing of *N* null hypotheses.

	Declared non-significant	Declared significant	Total
True null hypothesis	*TN*	*FP*	*N*_*0*_
False null hypothesis	*FN*	*TP*	*N—N*_*0*_
Total declared	*N—R*	*R*	*N*

*FP* and *FN* reflect number of false positives (Type I error) and false negatives (Type II error); *TP* and *TN* denote numbers of correctly declared significant and non-significant discoveries, respectively. Here *R* is the number of rejected null hypotheses (declared positives).

The proportion of errors committed by falsely rejecting null hypotheses can be viewed through the random variable $=\frac{FP}{FP+TP}$. FDR is the expectation of *Q*, and is controlled at a pre-specified threshold, *fdr≤q*, on an average [48].

$$FDR=E\left(Q\right)=E\left(\frac{FP}{FP+TP}\right)=E\left(\frac{FP}{R}\right)$$ (6)

The *q* level was selected to standard *α* level of 5%. For *N* multi-model testing correction (i.e. for three features analysed via two references, *N* = 18), the *p* values were arranged in ascending order, {*P(1)≤P(2)≤P(3)…≤P(N)*} corresponding to null hypotheses, {*H*_*1*_,*H*_*2*_,*H*_*3*_,*…*,*H*_*N*_}. Following this, in a step-up manner, the inequality given in Eq 7 was evaluated, in reverse sequential order, beginning from the last *p* value *P*(*N*),

$$P\left(i\right)\geq i\frac{q}{N}$$ (7)

The comparison was stopped when the above inequality was true. Finally, all the hypotheses {*H*_*i*_}_*i*=1..*k*_, for which *P*(*i*) was less than or equal to *P*(*k*) were rejected i.e., the models belonging to the rank *i*=1..*k* were significantly discriminant.

## 3. Results

All participants followed the cues and performed the tasks successfully. There was no feedback regarding fatigue during the experiment.

### 3.1 Time-frequency map

The TF maps were individually analysed for each participant during the trial period of -3 to 7 seconds, i.e. 10 seconds. Fig 3 shows TF maps of the representative three participants (participant 1, 2, and 4). The common average reference and the most reactive two bipolar channels (C3-Cz and Cz-C4) were selected for comparison, as discussed in section 2.5.1. The TF map of each participant was used for selecting reactive bands of ERD/ERS and peak latency from cue-onset (Table 2). Each feature pattern can be observed from Fig 3 and latencies in Table 2 (for all participants). For common average reference, participant 1, reflected a strong *beta* ERS on average between 19–27 Hz with peak latency from cue onset = 4.20–4.50 seconds *p*<0.05, during left and right KMI, respectively. Right KMI generated a concentrated *beta* ERS, compared to the left KMI. The bipolar method revealed a focused *beta* ERS at channel C3-Cz during left KMI i.e. no contralateral dominance. Contrary to this, channel Cz-C4 showed a prominent *beta* ERS during left KMI. For both references, *mu* ERD occurred at ≤ 12 Hz *p*<0.05 with participant 1. On average, the TF maps derived from both references demonstrated significant *beta* ERS for left and right KMI between 3.40–3.50 and 3.42–3.58 seconds, respectively. Similarly *mu* ERD was visible between 2.34–2.42 and 2.30–2.47 seconds respectively, and prominent *beta* ERD reflected between 1.87–1.91 and 1.93–1.89 seconds.

**Fig 4:**
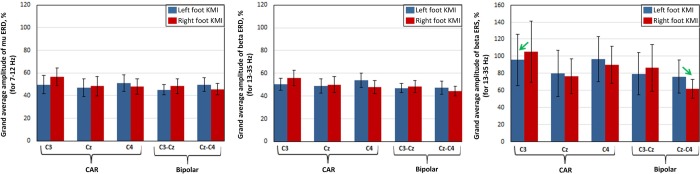
Average amplitude of significant *mu* ERD, *beta* ERD and *beta* ERS from all nine participants (N = 9). The blue bars show average amplitude of each feature after left foot task whereas red bars represent right foot task. The error bars depict standard deviations. The significant values of adjusted *p* < 0.017 are plotted.

Fig 4 reflects the grand-average amplitude of statistically significant *mu* ERD, *beta* ERD, and *beta* ERS for all nine participants. Since both low and high *beta* elicited ERD and ERS features, they are represented by *beta* (i.e. bandwidth of 13–35 Hz). For both references, each left-right feature amplitude exhibited differences. While *mu* and *beta* ERD, showed lower amplitude over both common average and bipolar references, the maximum amplitude was visible in case of *beta* ERS. It was also observed that common average reference channel Cz showed little difference in amplitude between left and right foot KMI. The common average channel C3 elicited strong left-right differences over the contralateral side for all three features, followed by C4 with relatively less strong contralateral dominance. Bipolar channel Cz-C4 reflected prominent contralateral difference in *beta* ERS discriminating the left-right foot KMI *p*<0.05, *t*-test). Maximum difference in amplitude between left and right KMI task is illustrated by green arrows in Fig 4.

### 3.2 Average time course of ERD/ERS

**Fig 5:**
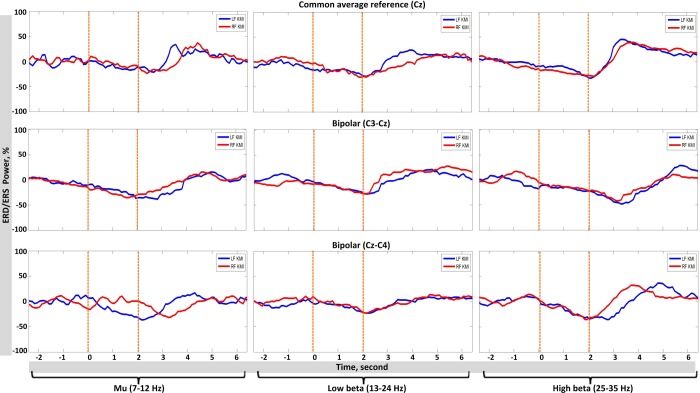
Average time course (participant 2) for ERD and ERS of common average reference channel Cz and two bipolar channels C3-Cz and Cz-C4 are shown. The left column reflects power changes in *mu* rhythm, mid column for *low beta* and right column for high *beta*.

Fig 5, represents the average time course of ERD/ERS for a representative participant (participant 2) with reference to the cue onset. The curves are based on most reactive ERD/ERS bands selected for each participant and peak latencies from cue onset, as reported in Table 2. The time-power curves of all participants reflected a strong peak in power amplitude at the end of the KMI period. The *mu* power attenuated with both common average and bipolar references, i.e. an ERD could be observed as soon as the visual cue was presented, with peak latency between 0–3 seconds, Fig 5. The low *beta* range also reflected an ERD from the beginning of the cue presentation window with a peak latency of 0.5–2.5 seconds. For high *beta*, a similar dip was observed during KMI period window i.e. approximately between 0–3.5 seconds, followed by a large spike i.e. an increase in power amplitude, ERS, on average of one second duration (post-imagery period).

### 3.3 EEG scalp topographies

**Fig 6:**
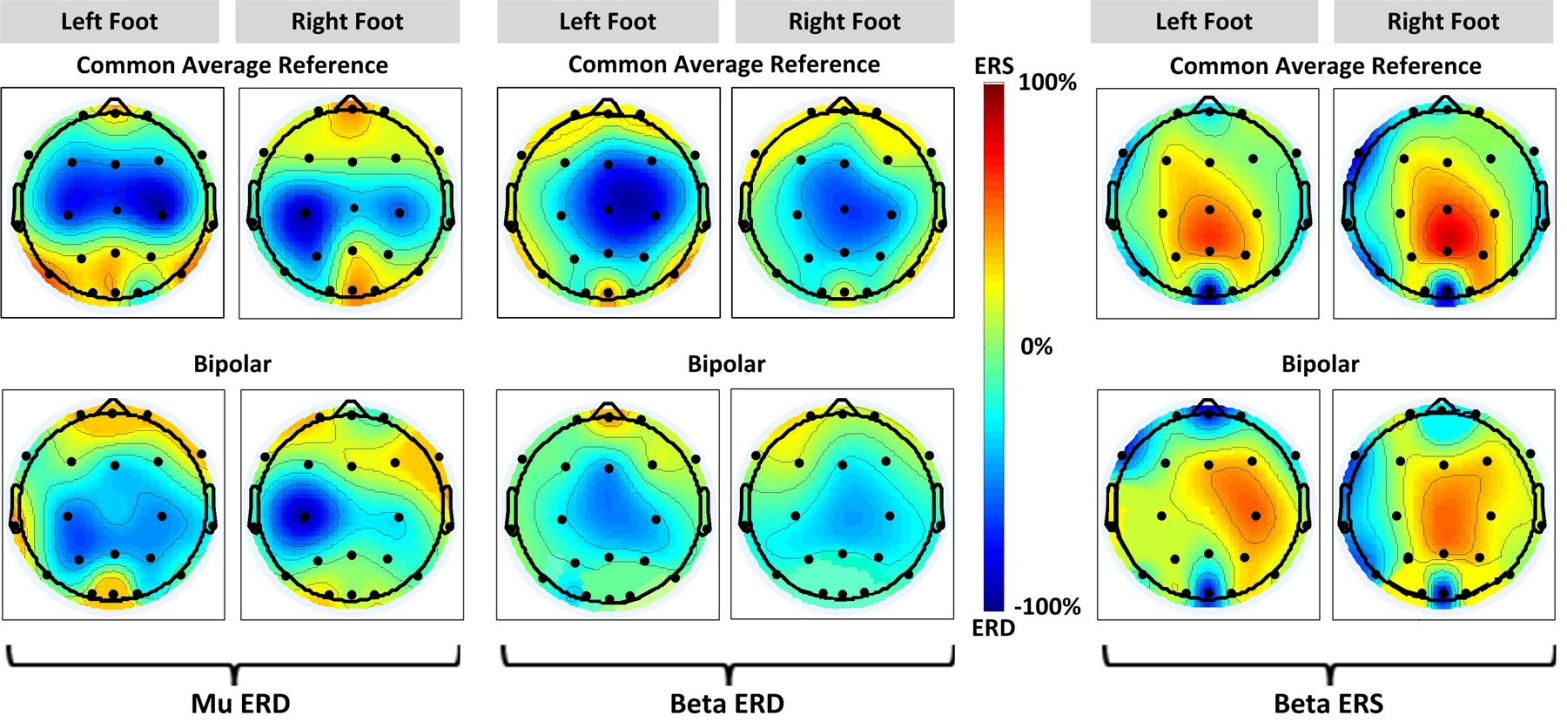
Average EEG topographies of ERD/ERS during foot KMI of all participants. Mu ERD is shown in the left column for left foot and right foot respectively, following this, *beta* ERD is in the mid column, and *beta* ERS is in the right column with distinguished ERS pattern. The top row illustrates ERD/ERS patterns for common average reference and the bottom for bipolar reference.

The EEG scalp topographies of ERD/ERS from all participants with their incidence time and average specific reactive bands are displayed in Fig 6. Low and high *beta* are represented by *beta* (13–35 Hz). We averaged the topographies over all nine participants. The common average reference method revealed that all three features were located across the vertex (Fig 6 top row). The *mu* ERD showed lateralized distribution during left and right foot KMI, whereas the *beta* ERS and ERD were localized centrally. Bipolar method (Fig 6 bottom row) demonstrated that *mu* ERD was contralaterally dominant during right foot KMI at Cz-C4, in agreement with results already obtained from Figs 3 and 4. Contrary to *mu* ERD, *beta* ERS revealed topographic scalp distribution with a contralateral dominance during left foot KMI at channel C3-Cz, whereas *beta* ERD remained centre-focused without lateralization. This is in accordance with our established findings from previous sections.

### 3.4 Classification accuracy

While in general, TF maps, average time-course for power, and scalp topographic analyses of the statistically significant EEG features revealed left-right KMI differences; there were instances (e.g. *beta* ERD amplitude graphs), where less differences exhibited. However, if a BCI user shows even a slight left-right difference, it is possible to enhance differences and improve the BCI control accuracy using machine learning techniques [50, 51]. To enhance the left-right differences and confirm the cortical lateralization of features, selection of classification method is critical. Classification for this research is derived from two linear i.e. LDA and SVM, and a non-linear model i.e. KNN.

Tables 4 and 5 show the classification accuracy of all EEG features, resulting from three models, for common average and bipolar references, respectively. With first reference, the highest accuracy was achieved by participant 1, 83.4%, for *beta* ERS during LDA classification. This was followed by SVM and KNN, i.e. 82.0% and 81.3% respectively. Maximum average accuracy with all models was observed for *beta* ERS compared to other features, given as KNN = 74.9% ± 5.20, LDA = 68.3% ± 6.72, and SVM = 67.2% ± 6.70. With bipolar reference, a similar trend was observed, i.e. participant 1 elicited highest classification accuracy of 80.4% for *beta* ERS using KNN. Similarly, average classification accuracies of *beta* ERS for all models was highest than the other features, i.e. KNN = 72.8% ± 3.64, LDA = 67.3% ± 4.70, and SVM = 65.7% ± 4.10. Results also reveal that the average accuracy of all features are well above the statistical chance level of a 2-class discrimination BCI problem which is 57.5% (*p*<0.05) or 60.0% (*p*<0.01) for 80 trials, as described in [52].

**Table 4:** The 5-fold cross-validation classification accuracy of left-right foot KMI using mu ERD, beta ERD, and beta ERS for common average reference.

Parti-cipant	Mu ERD						Beta ERD						Beta ERS					
	LDA		SVM		KNN		LDA		SVM		KNN		LDA		SVM		KNN	
	Acc (%)	AUC (%)	Acc (%)	AUC (%)	Acc (%)	AUC (%)	Acc (%)	AUC (%)	Acc (%)	AUC (%)	Acc (%)	AUC (%)	Acc (%)	AUC (%)	Acc (%)	AUC (%)	Acc (%)	AUC (%)
P1	69.2**	80.0	67.0**	79.0	65.3**	74.0	75.4**	85.0	72.0**	85.0	79.1**	85.0	83.4**	85.0	82.0**	82.0	81.3**	86.0
P2	74.0**	82.0	68.3**	79.0	60.2**	69.0	67.2**	80.0	61.2**	69.0	70.2**	81.0	73.5**	82.0	71.1**	81.0	74.2**	82.0
P3	64.2**	75.0	57.9*	66.0	59.4*	69.0	65.3**	80.0	64.4**	72.0	69.1**	81.0	64.2**	79.0	63.1**	69.0	66.2**	78.0
P4	69.1**	80.0	62.2**	72.0	61.0**	69.0	63.2**	69.0	62.2**	72.0	67.0**	79.0	67.4**	80.0	65.4**	72.0	76.4**	84.0
P5	65.2**	74.0	62.6**	73.0	57.5*	65.0	64.1**	80.0	61.1**	71.0	65.3**	81.0	62.5**	72.0	63.5**	71.0	71.2**	81.0
P6	66.3**	71.0	61.0**	69.0	68.1**	78.0	64.3**	79.0	60.5**	69.0	69.1**	84.0	67.7**	80.0	67.1**	79.0	69.1**	80.0
P7	64.0**	74.0	57.8*	66.0	66.1**	75.0	63.0**	79.0	58.0*	69.0	66.2**	77.0	68.0**	80.0	65.3**	72.0	78.3**	83.0
P8	68.0**	79.0	61.0**	69.0	57.6*	65.0	61.1**	69.0	57.5*	66.0	69.0**	83.0	61.0**	69.0	58.0*	68.0	77.0**	84.0
P9	67.1**	79.0	62.3**	72.0	62.2**	69.0	60.1**	79.0	59.0*	69.0	68.1**	79.0	67.0**	80.0	69.2**	80.0	80.4**	85.0
Mean	67.4	77.1	62.2	71.6	61.9	70.3	64.9	77.7	61.5	71.3	69.2	81.1	68.3	78.5	67.2	74.8	74.9	82.5
S.D.	3.10	3.69	3.60	4.84	3.90	4.44	4.50	5.30	4.30	5.45	4.01	2.57	6.72	4.95	6.70	5.53	5.20	2.55

* Over chance level of 2-class discrimination, 57.50% (*p* < 0.05).

** Over chance level of 2-class discrimination, 60.00% (p < 0.01).

**Table 5:** The 5-fold cross-validation classification accuracy of left-right foot KMI using mu ERD, beta ERD, and beta ERS for bipolar reference.

Parti-cipant	Mu ERD						Beta ERD						Beta ERS					
	LDA		SVM		KNN		LDA		SVM		KNN		LDA		SVM		KNN	
	Acc (%)	AUC (%)	Acc (%)	AUC (%)	Acc (%)	AUC (%)	Acc (%)	AUC (%)	Acc (%)	AUC (%)	Acc (%)	AUC (%)	Acc (%)	AUC (%)	Acc (%)	AUC (%)	Acc (%)	AUC (%)
P1	72.3**	81.0	68.2**	79.0	70.3**	81.0	61.1**	69.0	61.0**	69.0	64.2**	79.0	72.2**	82.0	70.0**	80.0	80.4**	85.0
P2	70.1**	79.0	67.3**	79.0	64.5**	76.0	67.3**	79.0	60.2**	71.0	69.0**	80.0	69.4**	79.0	66.0**	79.0	73.0**	81.0
P3	65.1**	78.0	57.6*	66.0	59.0*	71.0	62.2**	69.0	60.1**	69.0	70.0**	81.0	63.4**	69.0	61.0**	69.0	72.1**	82.0
P4	73.0**	82.0	62.2**	71.0	65.1**	79.0	65.3**	75.0	61.1**	79.0	60.3**	74.0	71.1**	81.0	70.1**	80.0	71.1**	81.0
P5	66.2**	78.0	60.1**	70.0	59.2*	71.0	63.0**	69.0	59.3*	68.0	68.1**	80.0	64.4**	76.0	66.0**	79.0	69.2**	80.0
P6	63.1**	69.0	61.1**	69.0	68.0**	77.0	57.5*	67.0	57.7*	67.0	71.1**	81.0	62.4**	72.0	60.1**	71.0	76.0**	84.0
P7	70.2**	79.0	59.0*	69.0	66.3**	80.0	58.4*	67.0	60.0**	69.0	64.2**	78.0	61.3**	69.0	61.0**	69.0	70.1**	81.0
P8	69.4**	79.0	58.0*	69.0	61.1**	71.0	67.2**	79.0	60.3**	80.0	74.0**	83.0	67.4**	78.0	69.0**	80.0	74.0**	83.0
P9	71.0**	80.0	62.3**	71.0	64.1**	77.0	69.1**	80.0	60.1**	79.0	69.0**	80.0	74.1**	83.0	68.0**	79.0	69.2**	81.0
Mean	68.9	78.3	61.8	71.4	64.2	75.9	63.5	72.6	60.0	72.3	67.8	79.5	67.3	76.5	65.7	76.2	72.8	82.0
S.D.	3.40	3.74	3.80	4.53	3.90	3.98	4.10	5.52	1.02	5.36	4.10	2.50	4.70	5.41	4.10	4.96	3.64	1.66

* Over chance level of 2-class discrimination, 57.50% (*p* < 0.05).

** Over chance level of 2-class discrimination, 60.00% (*p* < 0.01).

**Fig 7:**
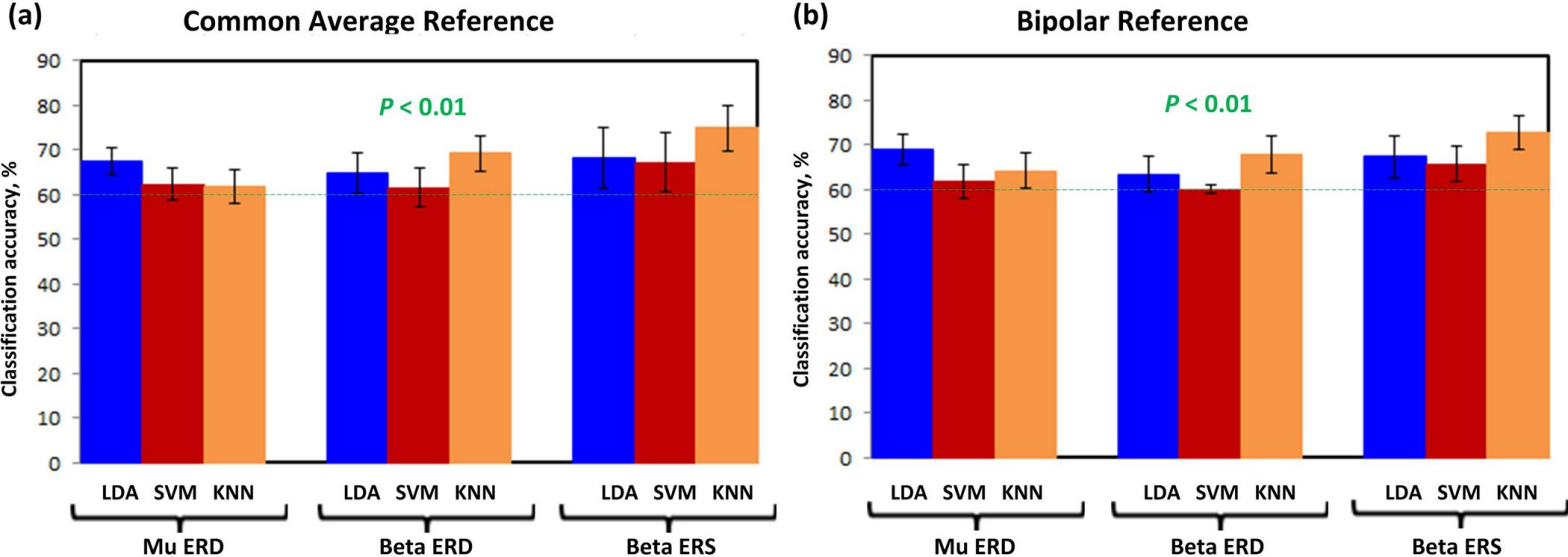
Classifiers performance accuracy in percentage, using (A) common average reference, and (B) bipolar reference. The error bars represent standard deviations.

In addition to classification accuracies, Tables 4 and 5 also reflect the area under ROC curve (AUC), in percentage, of each participant. For ideal detection AUC should be 100%. Beta ERS exhibited maximum AUC of 86.0% with KNN using common average reference, for participant 1. Similarly, with bipolar reference, it showed highest AUC of 85.0% with KNN model. Overall the average AUC was observed to be maximum for *beta* ERS using both references, i.e. KNN = 82.5% ± 2.55 and KNN = 82.0% ± 1.66, respectively. It can therefore be stated that *beta* ERS resulted in highest 2-class discrimination accuracy than other features, exceeding the chance level 60% at *p*<0.01, with highest AUC. However, *mu* and *beta* ERD also performed above the chance level, of 2-class discrimination, as described earlier (*p*<0.05 FDR adjusted). On average the KNN model outperformed LDA and SVM for all participants, as shown in Fig 7.

## 4. Discussion

This study focused on the analysis of *mu* and *beta* ERD/ERS, following left and right foot dorsiflexion KMI. The analysis comprised of TF maps, time-power ERD/ERS, and EEG scalp topographies, for common average and bipolar references. In general, a decrease in *mu* band activity (ERD) during KMI, with a significant increase in high *beta* band following KMI (ERS), was observed. Low *beta* ERD was observed during KMI with little post-KMI rebound, which was in agreement with literature [17, 53, 54]. Beta ERS was localized at the vertex i.e. the foot area representation of the cortex. With common average reference, *beta* ERS showed a high power concentration, whereas with bipolar reference low power concentration followed by contralateral dominance were observed at channels C3-Cz and Cz-C4, Fig 6. Mu ERD was distributed bilaterally across the vertex, in case of both references. Beta ERD was focussed at the vertex with no prominent lateralization. In general, the common average reference method resulted in strong reflection of ERD/ERS, which is evident in scalp topographies, Fig 6 as well as grand average amplitude graphs, Fig 4. The bilateral *mu* ERD and *beta* ERS, could provide a basis for left-right discrimination of KMI tasks, contralateral to the side of movement.

### 4.1 Common average reference and bipolar method for discrimination of left-right *mu* ERD, *beta* ERD, and *beta* ERS

The common average reference method has been used as it is computationally simple and compliant to both on-chip and real-time applications. This spatial filter identifies small signal sources in very noisy recordings, with much higher signal-to-noise ratio than a Laplacian [55, 56]. It was used to detect intention of movement during imagination [57]. In contrast to a previous study on left-right difference of *beta* ERS, which did not observe any difference with common average reference [17], our study confirmed the difference for all three features, not only *beta* ERS. Mainly, the channels adjacent to the vertex i.e., C3 and C4 exhibited a contralateral dominance with reference to cue presentation. The selection of these channels was based on the studies confirming that foot KMI elicits ERD/ERS patterns in the sensorimotor cortex [10, 24]. The analyses indicated that unilateral foot KMI generated significant *mu* ERD (*p*<0.01) and *beta* ERS (*p*<0.01) in all participants with no BCI feedback training. Our results depict that foot KMI elicits broad-banded ERD (10.1 Hz ± 1) and narrow-banded ERS (24 Hz ± 0.8). Highest 2-class discrimination accuracy was achieved for *beta* ERS and *beta* ERD features with this reference.

However, the transverse bipolar method also demonstrated statistically significant left-right discrimination of foot KMI with all features, maximum with *beta* ERS. In contrast to Laplacian, it is a simple spatial filter, that derives the first spatial derivative, thus enhances the differences in voltage-gradient in a direction [56]. With multiple comparisons of left-right features between both reference channels, the family-wise error rate Bonferroni correction was performed.

With foot KMI, the broad band ERD indicated the involvement of supplementary motor area (SMA) in the preparation and performance of imagery tasks [33, 58]. In addition to this, the foot area enhancement was observed with narrower *beta* ERS. Therefore, it can be stated that the differences in ERD and ERS can be observed in *mu*, low *beta* and high *beta* frequency bands of sensorimotor cortex and SMA. However, for one BCI system with multiple users, the use of all three features, as individual control signal, could be tricky, as the decision boundary/threshold to discriminate between the features (frequency band) may vary.

### 4.2 Comparison of KNN with linear classifiers using false discovery rate correction

Fig 7 depicts the results from present study (FDR adjusted *p*-values of 0.05) with an average classification accuracy of ≥60% (*p*<0.01) for all participants that deployed *mu* and *beta* ERD/ERS for discrimination between classes. It is evident for all three classifiers that *beta* ERS exceeded 80% accuracy for participant 1 (Table 4). These results clearly show an improved classification accuracy than a similar study [17], with the difference of BCI design. Our study involved artifacts rejection using ICA and a KNN as classifier model that outperformed remaining models for *beta* ERD and ERS, Fig 7. These efficient results could be due to the low dimensional feature vectors deployed in the study for a BCI [26].

Significant ERD/ERS features associated with *mu* and *beta*, were evaluated using AUC-ROC for the binary classifier. However, since each classifier model is independent, multiple comparisons between models were made to evaluate the statistical significance of the models. Consequently, the FDR correction was applied, using the method of Benjamini-Hochberg, as described in section 2.6.2. The threshold for controlling FDR was selected to standard *α* level of 5% for the purpose of comparison, *q*=0.050. Table 6 reflect the FDR corrections for LDA, SVM, and KNN models. It can be observed that all the comparisons resulted in the rejection of null hypotheses H_0_, except for the last comparison, where the inequality in Eq 7 becomes true. This is due to the very close accuracies exhibited by KNN and SVM models, i.e. the performance efficiency of both classifiers has similar impact for *mu* ERD. These encouraging results suggest that the foot dorsiflexion KMI can potentially elicit left-right differences in EEG. Following this, the feedback training plays an effective role in enhancing the classification accuracy as suggested by [59]. Subsequently, our next aim would be to monitor the repetitive use of BCI training and its effects on classification accuracy.

**Table 6:** False discovery rate (FDR) corrections for LDA, SVM and KNN classifiers.

			*p-*value	Linear setup	Adjusted-*p* q = 0.050	Rejected H_0_
Mu ERD	Common average reference	LDA-SVM	*	*	0.003	1
		LDA-KNN	0.007	*	0.006	1
		KNN-SVM	0.435	*	0.008	1
	Bipolar reference	LDA-SVM	*	*	0.011	1
		LDA-KNN	0.004	*	0.014	1
		KNN-SVM	0.028	*	0.017	1
Beta ERD	Common average reference	LDA-SVM	*	0.002	0.019	1
		LDA-KNN	*	0.003	0.022	1
		KNN-SVM	*	0.004	0.025	1
	Bipolar reference	LDA-SVM	0.011	0.007	0.028	1
		LDA-KNN	0.020	0.007	0.031	1
		KNN-SVM	*	0.010	0.033	1
Beta ERS	Common average reference	LDA-SVM	0.046	0.011	0.036	1
		LDA-KNN	0.007	0.020	0.039	1
		KNN-SVM	0.003	0.028	0.042	1
	Bipolar reference	LDA-SVM	0.041	0.041	0.044	1
		LDA-KNN	0.010	0.046	0.048	1
		KNN-SVM	0.002	0.435	0.050	0

* denotes the *p*-value less than 0.001.

Our next goal is to use the common spatial patterns (CSP) method for the same task i.e. discrimination of left-right foot KMI. In general, CSP has been used in BCI study for motor imagery (foot and hand/tongue) using only Laplacian derivation, not left and right foot KMI difference (e.g., [60]). However, our study reflected the enhancement of foot KMI differences using common average and bipolar references. The use of CSP features with the present study design, could improve the left-right differences of foot KMI and be used as independent control features.

## 5. Conclusions

The aim of this research was to decode the bilateral foot motor imageries and obtain high classification accuracy in order to enhance the universality of lower-limb assistive BCI. The results of presented investigation show the lateralization of *mu* and *beta* features in association with left-right foot dorsiflexion KMI. We have demonstrated that the KNN model, with common average reference method, can improve ERD/ERS lateralization. Our method achieved highest accuracy level of 83.4% using EEG signals from channels at the vertex and its adjacent C3 and C4 positions. It is therefore concluded that *beta* ERS, in addition to *mu* ERD, and *beta* ERD can be used as independent control features for a synchronous BCI. These features could be deployed in a 2-class BCI as control commands for operating bionic foot or a foot neuroprosthesis.

## Supporting information

S1 Data (XLSX)
